# Women with comorbid substance dependence and psychiatric disorders in Sweden: a longitudinal study of hospital care utilization and costs

**DOI:** 10.1186/s12913-015-0873-5

**Published:** 2015-06-06

**Authors:** Tina M. Olsson, Mats Fridell

**Affiliations:** Department of Social Work, Lund University, 220 00 Lund, Sweden; Department of Psychology, Lund University, 220 00 Lund, Sweden

**Keywords:** Cost of illness, Program evaluation, Treatment, Prevention, Intervention, Policy, Co-morbidity

## Abstract

**Background:**

Substance use disorders are regarded as one of the most prevalent, deadly and costly of health problems. Research has consistently found that the prevalence of other psychiatric disorders among those with substance related disorders is substantial. Combined, these disorders lead to considerable disability and health years lost worldwide as well as extraordinary societal costs. Relatively little of the literature on substance dependence and its impact on healthcare utilization and associated costs has focused specifically on chronic drug users, adolescents or women. In addition, the research that has been conducted relies largely on self-reported data and does not provide long-term estimates of hospital care utilization. The purpose of this study is to describe the long-term (24–32 year) healthcare utilization and it’s associated costs for a nationally representative cohort of chronic substance abusing women (adults and adolescents) remanded to compulsory care between 1997–2000 (index episode). As such, this is the first study investigating healthcare costs for women in compulsory treatment in Sweden.

**Methods:**

Women (*n* = 227) remanded to compulsory care for substance abuse were assessed at intake and their hospital care utilization was retrieved 5-years post compulsory care from national records. Unit costs for ICD-10 diagnoses were applied to all hospital care used from 1975–2006. Attempts are made to estimate productivity losses associated with hospitalization and premature death.

**Results:**

Upon clinical assessment it was found that a majority of these women had a comorbid psychiatric disorder (primarily personality disorder). The women followed in this study were admitted to hospital five to six times that of the general population and had stays six to eight times that of the general population. Total direct healthcare costs per person over the study period averaged approximately $173,000 and was primarily the result of psychiatric department visits (71 %) and inpatient treatment (98.5 %; detoxification and short-term rehabilitation).

**Conclusions:**

Women placed in compulsory care use more hospital resources than that of the general Swedish population and when compared to international research of hospital care use and substance abuse. Direct hospital costs vary greatly over the life course. Effective services can have significant economic benefit.

## Background

Substance abuse and dependence is one of the most prevalent, deadly, and costly of health problems [1–4] and is a component cause of more than 200 diseases in individuals leading to increased morbidity and mortality [1, 5–8]. In the United States, the economic cost of drug abuse has been estimated at $180.9 billion (2002 values; [9]) and the combined cost of alcohol and drug abuse has been estimated at $246 billion (1992 values) [10]. In Sweden, substance abuse costs society an estimated 2.65 billion US dollars (2008 values) [11].

Clinical, epidemiological and general population studies have found that comorbid substance use and psychiatric disorders are common in the general population and even more prevalent among treatment populations [12–15]. In Sweden, it is estimated that among substance abusing patients between 30 and 50 % suffer from a psychiatric disorder [11]. Mental illnesses [16], which include substance use and psychiatric disorders, are the leading causes of disability adjusted life years (DALYs) worldwide, accounting for 37 % of health years lost from non-communicable diseases [16]. In addition, mental and substance use disorders [2] were found to be the leading cause of years lived with disability worldwide with depression, substance use disorders, and anxiety accounting for 75 % of this burden [2]. Mental illness [17], including substance use disorders, rank in the top five of major non-communicable diseases worldwide with an estimated global cost to society at nearly $2.5 trillion – higher than that of cancer, cardiovascular disease, chronic respiratory diseases and diabetes - and a cost projected to surge to $6.0 trillion by 2030 [17].

Healthcare costs, along with crime costs and productivity losses, are consistently identified in international research as one of the largest contributors to the economic costs of substance abuse [9, 10, 18–22]. Despite the large economic burden to society of substance use and dependence as estimated by cost-of-illness studies, few studies have investigated the role of substance dependence in healthcare utilization or its associated cost. The growing literature suggests that substance use and dependence is positively and significantly related to health services utilization - such as hospital admissions and emergency room care - and cost [23–30]. Additionally, having a comorbid psychiatric disorder appears to increase the utilization and cost of healthcare services among patients with a primary diagnosis of substance use disorder [31].

Although the literature on substance dependence (and services) and its impact on healthcare utilization and associated costs continues to develop, relatively little of this research has focused specifically on chronic drug users [25], adolescents [32, 33], or women [29, 34]. In addition, longitudinal studies commonly have short (≤1 year) time frames and often rely on self-reported healthcare use data. Importantly, few studies formally assess the extent to which participants have a substance dependence diagnosis or a co-morbid psychiatric disorder (ICD; DSM-IV). Although exceptions to these identified limitations do exist [35–37], commentators interested in the future of research on substance abuse services have called for economic studies that focus on for example special populations of substance abusers [37]. These populations include for example women, adolescents and individuals with co-morbid disorders and which present long-term data [38].

Epidemiological data that describes healthcare utilization and costs over time for specific populations is valuable from a policy perspective as understanding trends in resource use can aid policy makers in decisions regarding how to target, deliver and improve services, as well as in understanding the potential benefits of effective intervention for specific groups of substance dependent individuals at specific points in time. Accurate empirical information could also help researchers in developing alternative models and formulating research initiatives. In addition, data regarding the longitudinal patterns of healthcare utilization and cost is of potential interest to researchers investigating the clinical outcomes of treatment services (such as reductions in healthcare utilization), as the majority of outcome studies do not include an economic arm [22, 39].

As such, the purpose of this study is to describe the long-term (24–32 years) pattern of healthcare utilization and its associated costs for a nationally representative cohort of chronic substance abusing women (adults and adolescents). These women were placed in compulsory care between 1997–2000 (index episode) as a result of their chronic substance abuse. Compulsory care in Sweden occurs when individuals are remanded to treatment, in an institutional setting, by the court even though he or she has not committed any crime. Although these women were originally identified, remanded to care and included in the cohort due to their substance use disorder, subsequent research [40–42] has found that 80 % of the patients had a comorbid substance dependence and psychiatric disorder (primarily personality disorder). Importantly, this is not a group of dual diagnosis patients [43]. That is, this group is not one characterized by severe mental illness but instead, is one of chronic substance abusing women with severe social problems, personality disorders, ADHD and persistent criminal behavior.

This study aims to answer the following questions:

1. What is the degree of resource use (number of hospitalizations and length of stay) by type of service among women remanded to compulsory care?
2. What is the total average direct cost of hospital care received by women remanded to compulsory care over the study period?
3. What is the value of lost productivity due to hospitalization and premature death for women remanded to compulsory care?
4. What is the potential benefit of preventing future hospitalizations among women remanded to compulsory care at a given age?

This study is the third in a series of economic studies describing this group. The first investigated the long-term pattern of criminal justice system involvement and costs [44] and the second investigated productivity losses associated with criminal behavior [45]. This study builds on these prior investigations by describing the group’s use of direct healthcare resources and associated productivity losses.

## Method

### Subjects

All patients admitted to Lunden from January 1, 1997 to December 31, 2000 (*n* = 227) were consecutively included in the cohort followed in this study. At the time of this study, Lunden was a 21-bed inpatient compulsory care residential treatment unit run by the National Board of Institutional Care (SiS). Lunden was one of 36 SiS run facilities nationwide with a national catchment area. The treatment unit was reserved for the treatment of women exclusively, with a focus on drug addiction. The unit staff included psychologists, psychiatrists, nurses, social workers, treatment attendants, and administration [46–48]. The institution had 9 beds for youth (LVU) and 12 beds for adults (LVM). In the Lunden cohort, 92 women (40.5 %), were remanded to LVU care and 135 (59.5 %) to LVM care. Thus, within the Lunden cohort, there are two distinct subgroups of patients (LVU and LVM).

A psychosocial assessment (SCID-II; DSM-IV) [49] was administered to all women at admittance and all participants met the diagnostic criteria for substance (narcotic) dependence. Additionally, substance use was assessed via supervised urine samples throughout index care episode and analyzed by Gas chromatography mass spectrometry (GC-MS). The substances used by the cohort prior to admission included: stimulants, mainly amphetamine (51 %), opiates, mainly heroin (35 %), alcohol (7 %), or sedatives/other drugs (<3 %). Cannabis was used by 20 % of the women in addition to other drugs [39–41]. This pattern of poly-substance use is common for clinical samples of narcotic drug abusers in Sweden [50].

The clinical assessment of psychiatric disorders was based on standardized diagnostic methods and psychological testing. It revealed that 78 % of the women had at least one personality disorder according to DSM-IV, SCID-II and 42 % had at least one psychiatric disorder. Psychiatric disorders (axis I; ICD-10) identified in this group included: anxiety- and depressive disorders (36 %), and psychoses (Schizophrenia 5 % and substance related psychosis 15 %) [40–42]. About 60 % of the patients had at least one prior serious suicide attempt.

The mean age of the subjects at intake to treatment was 18.7 years for the LVU subjects (range: 16 – 20) and 26.7 for the LVM subjects (range: 18 – 43) [40,41]. Of the women followed in this study, 92.9 % have been charged for at least one crime [44] and 22 died prior to five-year follow-up January 1, 2007 (4 LVU; 18 LVM) (9.7; 4.3 % LVU; 13.3 % LVM). In the cases of premature death, substance use was the primary cause of 18 deaths and somatic disease was the primary cause of four of the deaths. The causes of death were diagnosed by reviewing ICD-10 coding from autopsy protocols obtained from the forensic units in Sweden. In addition, death certificates were obtained from official records maintained by the National Board of Health and Welfare, Stockholm, Sweden and reviewed.

### The compulsory care system in Sweden

The compulsory care system in Sweden is charged with treating (1) youth with serious psychosocial problems and (2) adult substance abusers. Youth are admitted to compulsory care under The Care of Young Persons Act (LVU, act 1990:52). According to the LVU, “A care order is to be issued, if the young person exposes his health or development to a palpable risk of injury through the abuse of addictive substances, criminal activities, or some other socially degrading behavior.” (LVU, act 1990:52, section 3). Adult substance abusers are admitted to compulsory care under the Law on Compulsory Care for Substance Abusers (LVM, act 1988:870). Under Section 4 of the LVM, a court can order compulsory care for a person whose health is deemed to be at risk, or who may be placing others at risk, and who is considered to need assistance in order to discontinue substance use. The LVU and LVM acts are unrelated to penal code or laws of psychiatric care. Individuals exhibiting a need for compulsory care are primarily reported to the court by: social welfare, police, family members, psychiatrist, substance abuse treatment provider or, more rarely, a general practitioner. Within 8 days of the report, an assessment of need for treatment or physician evaluation must be completed, and court hearings proceed. For those remanded to compulsory care, care orders are implemented in specially certified LVU and LVM facilities that are run under the authority of the National Board for Institutional Care (SiS). The compulsory care system is separate and independent from the mainstream healthcare system in Sweden. The number of women undergoing compulsory care annually in Sweden is approximately 650 – 700 [51].

### Consent

Written consent to participate in the present study was provided by all participants. Participation in the present study was voluntary and individual participants could drop out of the study at any time. Information was provided verbally and in written form to individuals prior to recruitment as well as prior to follow-up, five years post referral. Participants provided written consent at both of these time points.

### Economic analysis

#### Resource use

The National Patient Register (NPR) maintained by the National Board of Health and Welfare, Stockholm, Sweden provides the basis for resource use measures. Data on healthcare resource utilization at the individual level is collected prospectively for all Swedish citizens and housed in the NPR. NPR data was accessed retrospectively, five-years post index care admission. Data spans the period 1975, when the NPR was introduced in Sweden through 2006, 5-years following the last index care admission. As individuals entered the study at varying ages, healthcare resource use was tracked for between 24 and 32 years depending on the age of the participant when they entered compulsory care. All in hospital outpatient physician visits and inpatient hospitalizations including length of stay and primary diagnosis (ICD) were included in the NPR for the period under review.

#### International classification of diseases (ICD)

During the period under review, primary diagnosis was coded in the NPR using three versions of the ICD: 1975 to 1986, ICD-8; 1987 to 1996, ICD-9; and 1997 onward, ICD-10. In order to align the ICD coding with the unit cost coding, all codes were converted to ICD-10 equivalents or approximates using primarily three documents [52–54] and one on-line resource (www.icd10data.com). Conversion was made by manually comparing text from the diagnoses coded with ICD-8 and ICD-9 to the text of ICD-10 codes.

#### Unit cost

Unit cost estimates were applied at the individual level to all hospital visits. Costs per hospital visit were summed to arrive at a total cost per person.

#### Cost Per Patient Database (KPP)

Unit costs per ICD-10 primary diagnoses were taken from Sweden’s Cost per Patient Database (KPP) maintained by the Swedish Association of Local Authorities and Regions [55, 56] and publicly available. The KPP provides nationwide estimates of unit costs within the hospital setting. These cost estimates are based on the costs of providing services and estimation is based on individual patient contacts with hospital care across Sweden. The KPP provides a nationwide estimate of the cost per visit based on ICD-10 diagnoses and includes main diagnosis and all subsequent diagnoses and is not adjusted for co-morbidities. In this analysis, the 2010 unit (day) cost for the average woman receiving care by primary diagnosis was used to value hospital stays.

#### Outpatient visits

In most cases the cost per outpatient visit was not available. For all outpatient physician visits the daily rate of inpatient hospitalization by primary diagnosis is used as a proxy for the cost per outpatient physician visit.

#### Productivity loss

There are several methods available for estimating productivity loss [57, 58]. In this study, productivity loss is measured using the human capital approach [59] and as such follows the methodology used in two prior studies of this group [44, 45]. This approach values productivity loss as the present value of lost time according to the market wage. Productivity loss due to hospital stays is valued using the average wage for women during 2010 of 26,200 SEK ($2,901) per month [60]. Lost life is valued using the present value of future earnings and estimated using the average monthly wage for women across Sweden by age (ibid).

#### Currency, inflation and time preference

Costs were calculated in Swedish crowns (SEK) and are reported in the text and tables in US dollars (2010). Unit costs were estimated for year 2010 and applied at the individual level to resource use. The purchasing power parity (PPP) based exchange rate in 2010 was 9.03 SEK = 1.00 US Dollar = 0.76 Euro [61] and was used to convert currency in this study. All currency conversions were conducted post-analysis.

In the primary analysis, no discounting is performed. However, discounting is performed in subsequent analyses order to show how effective prevention at various time points (ages) could impact direct healthcare cost outcome. Prior studies of this population have found that depending on substance of choice (i.e., alcohol, cocaine, heroin, etc.) and group (i.e., LVU, LVM), debut age for substance use occurred between the ages of 12 and 20. In addition, the women in the LVU group were placed in index care between the age of 17 and 19 while the women in the LVM group were placed in index care between the ages of 21 and 30 [40]. These age points are used as probable intervention points in order to estimate the present value of potential benefits of prevention activities. Additionally, discounting is performed in order to estimate the present value of productivity loss at (1) time of first hospitalization and (2) time of death.

#### Missing values

In the original data set, 116 (2.4 %) entries either did not provide a primary diagnosis or the primary diagnosis code given did not correspond with a diagnosis code from ICD-8, ICD-9 or ICD-10. These entries were subsequently removed from the analysis.

#### Statistical analysis

All primary data is housed in Excel version 14.0.0 and all analyses in which discounting is performed were conduced in Excel. All other analyses were conducted using SPSS version 20. Descriptive statistics are presented for the entire sample (Lunden) as well as for the subgroups found within the sample (LVU, LVM) separately. Comparisons are not made between groups.

## Results

### Hospital admissions

**Fig. 1:**
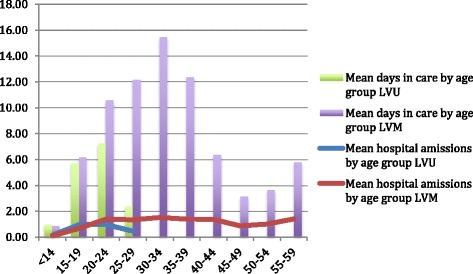
Resource use: Mean number of hospital admissions and mean number of days in care per person per year by age group based on number of participants at age

During the period under review, women were admitted 4,737 times for inpatient hospitalizations (87.9 %), outpatient visits (10.3 %) or emergency care (1.6 %). Individuals were admitted most often, 41.8 % for psychiatric department visits. These were followed by: somatic care other than reproductive health (ob/gyn) (29.4 %), alcohol/drug services (20.2 %) and ob/gyn visits (8.3 %). The mean number of hospital visits per person over the study period was 20.87 (12.65, LVU; 26.47 LVM) (sd, 30.87 Lunden; 15.14 LVU; 37.06 LVM). Table 1 reports the average number of hospital admissions per person. Figure 1 shows the average number of hospital admissions per year at a given age over the life course.

**Table 1:** Resource use: average (standard deviation) number of hospital visits and length of stay (days) per person by primary service for the Lunden cohort and the LVU and LVM subgroups

	LVU *n* = 92		LVM *n* = 135		Lunden *n* = 227	
	Mean (s.d.) hospitalizations	Mean (s.d.) length of stay	Mean (s.d.) hospitalizations	Mean (s.d.) length of stay	Mean (s.d.) hospitalizations	Mean (s.d.) length of stay
*Clinic*						
Alcohol/drug services	2.67 (7.38)	12.76 (47.27)	5.28 (8.08)	25.4 (41.2)	4.22 (7.9)	20.27 (44.1)
Psychiatric department	4.01 (6.68)	52.46 (142. 48)	11.96 (24.64)	171.56 (519.83)	8.74 (19.8)	123.29 (414.52)
Reproductive health	.96 (1.56)	3.1 (6.43)	2.29 (3.51)	9.2 (16.52)	1.75 (2.95)	6.73 (13.69)
Other Somatic	4.98 (6.33)	15.29 (33.47)	6.93 (10.21)	26.08 (41.74)	6.14 (8.88)	21.70 (38.89)
*Type of Admission*						
Inpatient	10.86 (13.17)	83.50 (167.70)	23.44 (32.91)	231.74 (543.22)	18.34 (27.39)	171.66 (437.73)
Outpatient	1.62 (2.42)	-	2.54 (4.38)	-	2.17 (3.73)	-
Emergency	.14 (.45)	-	.47 (1.12)	-	.34 (.92)	-
Total	12.65 (15.14)	83.63 (167.80)	26.47 (37.06)	232.24 (543.50)	20.87 (30.87)	172.01 (437.98)

### Length of stay

The average number of days in care per person during the period under review was 172.01 (sd 437.98) for the Lunden cohort and 83.63 (sd 167.80) and 232.24 (sd 543.50) days for the LVU and LVM subgroups respectively. The average LOS per person per hospital admission was 8.24 (sd 35.64) for the cohort (LVU 6.61, sd 24.08; LVM 8.77, sd 38.65). Table 1 reports the average length of stay per person. Figure 1 shows the average number of days in care per year at a given age over the life course.

### Total costs

**Fig. 2:**
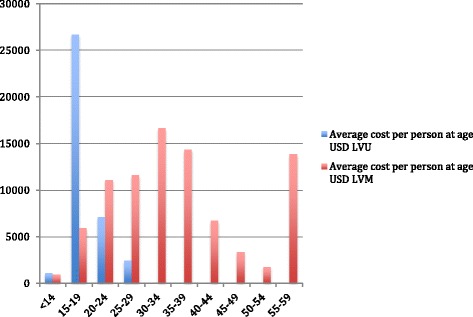
Mean direct hospital care cost per person per year by age group based on number of participants at age

Table 2 reports the average total direct hospital costs per person by clinic and admission type. Average total direct hospital costs per person were $173,048 ($82,857, LVU; $234,512 LVM) (sd $446,009 Lunden; $166,911, LVU; $554,246 LVM). Psychiatric department visits made up the majority of total costs (70.8 %). These were followed by: somatic care other than ob/gyn (13.2 %); alcohol/drug services (12 %); and ob/gyn visits (3.8 %). Hospital costs were primarily the result of inpatient treatment (98.5 %). Figure 2 shows the average cost per person by age over the life course.

**Table 2:** Healthcare costs: average (standard deviation) total direct hospital care costs per person by primary service for the Lunden cohort and the LVU and LVM subgroups, USD 2010

	LVU *n* = 92 mean (s.d.) cost (USD)	LVM *n* = 135 mean (s.d.) cost (USD)	Lunden *n* = 227 mean (s.d.) cost (USD)
*Clinic*			
Alcohol/drug services	14,341 (68,866)	25,387 (43,822)	20,911 (55,478)
Psychiatric department	50,012 (129,941)	127,791 (529,944)	122,612 (420,622)
Reproductive health	2,755 (6,034	9,224 (16,445)	6,602 (13,607)
Other Somatic	15,749 (38,333)	27,810 (44,945)	22,922 (42,716)
*Type of Admission*			
Inpatient	81,209 (165,568)	231,419 (550,841)	170,541 (443,179)
Outpatient	1,474 (2,394)	2,501 (4,316)	2,085 (3,689)
Emergency	173 (766)	592 (1,514)	422 (1,280)
Total	82,857 (166,911)	234,512 (554,246)	173,048 (446,009)

Table 3 summarizes the average value of lost productivity per person due to hospitalization and premature death. The average age of death for the 22 (4 LVU, 18 LVM) women that passed away prior to 2008 was 34.68 (sd 10.53) (LVU 21.25, sd 3.20; LVM 37.66, sd 9.13). Premature death accounted for the largest proportion of productivity losses.

**Table 3:** Present value of lost productivity due to hospitalization and premature death for the Lunden cohort and the LVU and LVM subgroups USD, 2010

	LVU *n* = 92 Mean (s.d) cost (USD)	LVM *n* = 135 Mean (s.d.) cost (USD)	Lunden *n* = 227 Mean (s.d.) cost (USD)
*Hospitalization*			
0 %	7,974 (16,000)	22,144 (51,822)	16,401 (41,761)
3 %	6,412 (13,196)	15,751 (35,991)	11,966 (29,313)
5 %	5,659 (11,850)	12,878 (28,995)	9,952 (23,825)
*Premature death*			
0 %	66,112 (312,222)	131,229 (354,362)	104,838 (338,722)
3 %	36,702 (173,103)	88,568 (232,333)	67,547 (211,475)
5 %	26,823 (126,504)	71,372 (185,383)	53,317 (165,230)

Table 4 reports on the present value (direct hospital costs) of preventing hospitalizations at various time points in an individual’s life.

**Table 4:** Present value of preventing future hospitalizations at given age for the Lunden cohort and the LVU and LVM subgroups, direct hospital costs, USD (2010)

	LVU *n* = 92 mean (s.d.) cost (USD)	LVM *n* = 135 mean (s.d.) cost (USD)	Lunden *n* = 227 mean (s.d.) cost (USD)	LVU *n* = 92 mean (s.d.) cost (USD)	LVM *n* = 135 mean (s.d.) cost (USD)	Lunden *n* = 227 mean (s.d.) cost (USD)
Age	3 % Discount			5 % Discount		
13	56,892	151,373	113,581	49,868	119,166	91,447
14	57,616	153,617	115,217	51,357	122,783	94,213
15	55,645	155,180	115,366	50,153	125,818	95,552
16	54,946	115,472	115,650	50,247	128,320	97,091
17	52,816	157,483	115,627	48,907	131,351	98,374
18	46,087	159,310	114,021	42,878	134,966	98,131
19	43,083	157,182	111,543	40,550	134,673	97,024
21	33,676	147,688	102,086	32,263	128,805	90,188
26	2,382	104,626	63,729	2,341	93,960	57,313
27	1,405	98,268	59,523	1,389	88,977	53,942
29	--	82,162	49,297	--	75,356	45,214
30	--	74,736	44,842	--	69,042	41,425

## Discussion

The purpose of this study was to describe the long-term (24–32 years) healthcare utilization and its associated costs for a nationally representative cohort of chronic substance abusing women in compulsory care. The participants in this study had on average over 20 hospital admissions with total average days in care of 172 per person (Table 1) over the study period. This resource use varied greatly by group and age over the life course (Fig. 2). National figures [62] stemming from population based healthcare use report the average number of healthcare visits (including primary care) in 2010 to be approximately 15.4 per 100. Depending on age, the participants in this study were admitted to hospital five to six times (65.2-86.9 admissions annually per 100) more than the general population. Similarly, the total number of days in care per person across the population in 2010 was reported at 88 per 100 [63]. Participants in this study averaged six to eight times (537–716 days annually per 100) the national average. These results indicate that women placed in compulsory care for substance abuse have a substantially higher rate of hospital admissions and extended length of stays compared to that of the general Swedish population.

Not only did the participants in the current study use more hospital care resources as measured by hospital admissions and length of stay but they also appear to use more resources when compared to international research on healthcare utilization and substance use. For example, in a nationally representative study on illicit drug use and health services utilization [23], female participants’ (aged 18–60; substance using and non substance using) were hospitalized on average 0.131-0.171 times and visited the ER on average 0.373-0.429 times during the year. Estimation results for count measures of healthcare utilization revealed that heavy drug users had about a 30 % higher rate of hospital admissions than non-drug users. Similarly, in a study undertaken by French and colleagues [25] which estimated the annual use of hospital care, chronic drug users and injection drug users (men and women) were admitted to hospital 0.31 and 0.32 times respectively. Further, outpatient visits and emergency room episodes ranged from 1.23-1.42 and 0.78-0.79 respectively for the two groups of drug users.

The inflated resource use as found in this study translated to hospital care costs for women remanded to compulsory care for substance use much higher than that of the general population. The average direct cost of hospital care per person was approximately $173,000 over the study period (Table 2). The annual value of direct hospital care costs varied greatly depending on age and group (Fig. 2). National figures [64] place the average cost for healthcare (including primary care and pharmaceuticals) across the population at approximately $2,300 per person in 2010. The participants in this study had an estimated annual cost of hospital care between approximately two and three times this national average. Further, psychiatric care made up approximately 9 % of the national average while in this study, psychiatric care accounted for approximately 71 % of the total healthcare costs incurred by this group. Lost productivity due to hospitalization and premature death added substantially to this average cost and increased total cost estimates by 70 %.

It is clear that effective services for chronic substance abusing women can have significant economic benefits. A recent review which included twenty-four research reviews and forty-three research trials of evidence-based treatments for substance abusing patients with comorbid psychiatric disorders [63] found that the effectiveness of treatment varies by type (combination) of co-occurring disorder [65]. Further, depending on the nature of the diagnosis, stability in recovery may be contingent upon addressing the substance abuse early on while improvement of non-substance related disorders may precede improvement of substance use symptoms. Generally, treatment planning for patients with comorbid substance use and psychiatric disorders is more effective when effective therapeutic modalities (i.e., psychotherapy, pharmacotherapy and behavioral treatments) are combined [66, 67]. Unfortunately, none of the research reviewed assessed changes in patient hospitalization. Further research on the clinical outcomes of interventions for individuals with comorbid substance dependence and psychiatric disorders should attempt to incorporate resource use measures into assessment batteries [66].

It should be highlighted that a whole 10 % of the women in this study died prematurely at the average age of 34.5 years, within five years of index care episode. This is much earlier than was found in two prior studies conducted on substance abusing women in Sweden, which found the average age of premature death to be 37.5 and 42 years [7, 67].

## Conclusions

It is clear that women remanded to compulsory care for substance abuse are an extremely vulnerable group, even when compared to other populations of substance dependent individuals in Sweden and internationally. A majority of women remanded to compulsory care for substance abuse have comorbid psychiatric disorders with multi-problem profiles including a high rate of personality disorder. In addition, their use of hospital care as measured by episodes of hospitalization and length of stay is substantially higher than that of the general public. The healthcare system that can positively impact the extent, to which this population is hospitalized, especially in inpatient psychiatric care, can not only improve the lives of substance abusing women but can also improve the healthcare system. This represents an opportunity for policy makers within psychiatric and compulsory care to improve the health monitoring and treatment of patients with comorbid substance abuse and psychiatric disorders. The ability of the healthcare system as well as the compulsory care system to positively impact health outcomes for this group is pressing as a whole 10 % of participants died prematurely -- less than five years following compulsory care placement.

This study provides long-term individual based estimates of the potential benefit of effective prevention activities for reducing hospitalizations among women with comorbid substance use and psychiatric disorders. This gives policy makers an estimate by which to compare the costs and effects of intervention activities. For example, an intervention that targets this population at 16 years of age, costs $10,000 per person and has the potential of preventing hospitalizations over a two-year period in 10 % of the individuals treated, may provide close to $10,000 in net benefit (Table 4). In other words, when policy makers know the cost and potential effect of competing interventions, the estimates provided in this study can be used to estimate the potential healthcare benefit of these interventions. It is, however, necessary for studies investigating the effects of prevention and treatment activities to routinely assess the extent to which substance abusing women have a comorbid substance use and psychiatric disorder as well as track changes in economic variables such as criminal behavior, hospitalizations and employment patterns. This does not happen routinely in current research initiatives.

### Methodological considerations and study limitations

The costing methodology used in this study has advantages over other approaches as it (1) followed a nationally representative sample of substance abusing women in compulsory care; (2) employed standardized diagnostic assessment instruments; (2) used actual resource use based on national records as opposed to self reports; and (3) used national unit cost estimates. Other unique aspects of this study include the assessment of long-term (24–32 years) costs and the inclusion of both adolescent and adult women. The breakdown of healthcare costs by age and inclusion of productivity losses facilitates the use of these estimates for a wide variety of research, evaluation and policy purposes. Still, several methodological issues should be highlighted.

First, outpatient physician visits in this study were valued using the average cost per day for hospitalization, as costs per outpatient physician visit were not available. This is likely an overestimate of the cost of outpatient care as hospital stays include for example hoteling, which is not part of the cost of outpatient care. The estimate produced, however, accounted for a small portion (1.2 %) of the direct healthcare costs estimated in this study. Further, the average cost per outpatient physician visit in this study was valued at approximately $997 per visit. This falls within the estimate of $111 – 2,879 (2001 values) per outpatient physician visit found in a prior study of costs within the hospital setting [68].

Second, the cost of primary care was not valued in this study. This data is not routinely reported in Swedish registers and was therefore not available for this study. However, this group of patients in general is not treatment seeking and the individuals are chronic non-compliers; hence, when appointments are made, they are often missed. For the purposes of this study, therefore, the cost of primary care for this group may be negligible.

Third, in this study, pharmaceutical use was not valued. Although estimates are available for pharmaceutical use among the general (female) population [69, 70] and among patients in outpatient psychiatric care [71] in Sweden, no estimates are available for pharmaceutical use among chronic substance abusers or among patients with co-occurring substance use disorders. As the clinical study upon which this study is based did not track pharmaceutical use we were unable to base this estimate on primary data and it is uncertain as to how accurate the above estimates are for this population.

Fourth, the cost of sick leave absence and presenteeism was not estimated in this study as access to primary data on sick leave for this sample was not available. Although estimates on sick-leave absence among the general population are available [72] it is uncertain how accurate these estimates are for this population.

Fifth, the cost of informal care was not included in this study. Informal care occurs when people, without payment, provide help and support to family members or friends who may not be able to manage without this help because of frailty, illness, or disability. Studies have shown that carers of persons with a severe mental illness or co-occurring disorder provide substantial support in terms of time spent providing care, involvement in crisis situations and monetary expenditures. For example, a study of families of individuals with a substance use disorder found that 89 % of the families in the study provided informal care. On average, families in the study provided between 16.72 and 38.75 h of informal care during a two-week period [73]. Another study found that on average primary caregivers provided 73 more hours of care each year than non-primary caregivers [74]. The group of women followed in the present study, however, often lacked close relatives and therefore had few contacts with relatives or informal caregivers [40].
